# The permissive binding theory of cancer

**DOI:** 10.3389/fonc.2023.1272981

**Published:** 2023-11-09

**Authors:** Caroline M. Weisman

**Affiliations:** Lewis-Sigler Institute for Integrative Genomics, Princeton University, Princeton, NJ, United States

**Keywords:** cancer evolution, invasion and metastasis, protein interactions, cancer epigenetic evolution, theory of cancer, evolutionary mechanism

## Abstract

The later stages of cancer, including the invasion and colonization of new tissues, are actively mysterious compared to earlier stages like primary tumor formation. While we lack many details about both, we do have an apparently successful explanatory framework for the earlier stages: one in which genetic mutations hold ultimate causal and explanatory power. By contrast, on both empirical and conceptual grounds, it is not currently clear that mutations alone can explain the later stages of cancer. Can a different type of molecular change do better? Here, I introduce the “permissive binding theory” of cancer, which proposes that novel protein binding interactions are the key causal and explanatory entity in invasion and metastasis. It posits that binding is more abundant at baseline than we observe because it is restricted in normal physiology; that any large perturbation to physiological state revives this baseline abundance, unleashing many new binding interactions; and that a subset of these cause the cellular functions at the heart of oncogenesis, especially invasion and metastasis. Significant physiological perturbations occur in cancer cells in very early stages, and generally become more extreme with progression, providing interactions that continually fuel invasion and metastasis. The theory is compatible with, but not limited to, causal roles for the diverse molecular changes observed in cancer (e.g. gene expression or epigenetic changes), as these generally act causally upstream of proteins, and so may exert their effects by changing the protein binding interactions that occur in the cell. This admits the possibility that molecular changes that appear quite different may actually converge in creating the same few protein complexes, simplifying our picture of invasion and metastasis. If correct, the theory offers a concrete therapeutic strategy: targeting the key novel complexes. The theory is straightforwardly testable by large-scale identification of protein interactions in different cancers.

## 1 Introduction

### 1.1 The puzzle of invasion and metastasis

**Figure 1:**
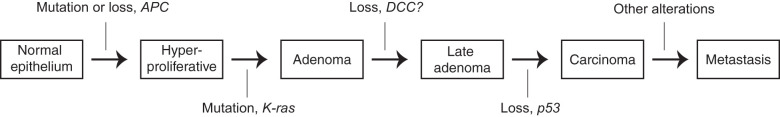
A simplified version of the schematization of the mutation-centric theory, introduced in (2), which proposes that the successive accumulation of genetic mutations causes the changes of early oncogenesis. The figure depicts the particular mutations that, under this theory, prompt progression from one stage of cancer to the next in colorectal cancer.

The evolution of cancer can be conceptually divided into early and late stages. In the early stage, cells proliferate excessively *in situ*, forming a primary tumor mass. There is now general agreement on what causes this process: genetic mutations. Reproducible “driver mutations” accumulate sequentially, pushing cancer development forward with each hit. These drivers affect genes in a finite number of pathways whose identities – for example, cell proliferation and apoptosis – are well-suited to explain the phenotypes, like excessive growth, observed in these stages of cancer (1, 2): they require only increases or decreases in their normal functions, which were usually already in operation to some degree in the ancestral cell. This picture constitutes a general explanatory framework for cancer evolution, which I will refer to as the “mutation-centric” framework. It was formally introduced in 1988 in a key paper by Kurt Vogelstein (2) (there referred to as the “genetic model”), whose central figure which I have mildly simplified as **Figure 1** below.

The mutation-centric framework is not complete, but is compelling and robust: it is consistent with much of the existing data, and is readily able to explain its major features. When one uses it to consider questions raised by early cancer evolution, one feels that one is approaching the problem in more or less the right way.

In later stages of cancer evolution, cells evolve to invade surrounding tissue, migrate, and colonize new sites in the body, where they form metastases. During this “invasion-metastasis cascade,” cells evolve a successive series of abilities that they previously entirely lacked. They break free of epithelia; push past neighboring cells; move into and out of vasculature; survive transport in circulation; and, finally, reach new tissues, only then beginning to adapt to the many new demands of its radically foreign ecology. Colonization is particularly baffling (3), as it seems to require many distinct adaptations, one for each feature of the new environment to which it is not initially suited, that do not seem to have anything to do with one another, or with the adaptations gained in earlier stages (4). Perhaps most remarkable is that cancer passes through each of these life stages in turn, acquiring radical new abilities and forms only briefly before moving on to the new set required by its next phase. How invasion and metastasis happen is a profound question for evolutionary biology.

One is naturally inclined to consider the invasion-metastasis cascade through the mutation-centric framework in trying to understand it. But, even since the introduction of the framework in 1988, as indicated by the striking comparative vagueness of the final arrow in **Figure 1** (identical to the original), this has proved largely unsuccessful. Searches for recurrent driver mutations, akin to those found in the early stages, have failed (1, 3, 5–7). Worse, metastases do not even seem to require new mutations relative to their primary tumor counterparts (8, 9), directly contradicting the main tenet of the framework. Beyond the framework’s empirical failures, invasion and metastasis present it with conceptual problems. It is not clear how it can accommodate, let alone explain, some basic phenomenology: cancer stem cells, and their relationship to invasion and metastasis (3, 10, 11); polyploid (12) and polyaneuploid (13) giant cancer cells; dramatic genome perturbations like aneuploidy (14) and chromothrypsis (15); and cancer reversion following movement to a normal environment (16–18) or transient reversion of driver mutations (19) It also seems, a priori, a poor match for the evolutionary problem. Many seemingly distinct adaptations are required; if each requires even a few mutations, the total number seems too large to be possible. In brief, the mutation-centric framework, though not strictly disproven, just does not feel like a good fit.

What one considers to be the most explanatorily useful entity in considering a problem is among the most important choices in a scientific enterprise. The correct choice allows discovery of fundamental principles; the wrong one all but precludes it. Is there a type of molecular change that could do better than mutations in explaining observations to date, in guiding future investigation, and in presenting unifying principles through which what is now a pile of disjointed facts may appear a coherent whole? In light of the limitations of the mutation picture, it would be foolish not to search for such alternatives. Indeed, many have already offered proposals (20). Here, I do the same.

### 1.2 A “binding-centric” framework: the permissive binding theory of oncogenesis

Here, I propose a general molecular mechanism underlying cancer evolution. It was conceived of to address specifically the conceptual gap posed by the later stages of invasion and metastasis, but it may also apply just as well to components of the early ones. In brief, the theory holds that protein binding is substantially permissive, rather than mainly instructive, as we often think. Natural selection has acted (either “intentionally” or as a side effect of other actions) to *limit* binding interactions, pruning many that exist in a baseline state in which they are abundant. Perturbations to cells may generate configurations of proteins that have not been effectively subject to this pruning. These are in the baseline, “permissive state,” such that many novel protein binding interactions are unleashed. Significant physiological perturbations are ubiquitous in cancer, and especially in its later stages, where they unleash many new binding interactions in this way. A subset of these interactions happen to cause functions that the nascent cancer finds useful; these drive oncogenesis.

Binding interactions are central in the theory, taking the place of mutations as the key entity with both causal and explanatory power. These include interactions between pairs of proteins, but also between proteins and DNA (as transcription factors). They are the direct causal effectors of oncogenesis, as protein interactions are for most biological functions. They are *not* the ultimate cause of oncogenesis: they are determined by a large number of more fundamental physiological factors, like the cell’s physical environment, chromatin state, genome, gene expression state, and so on. With perfect knowledge, we would be able to explain all protein interactions in the cell as a function of these underlying factors. But because the relationship between these levels is enormously complex, this ability is unlikely to exist for many years. In its stead, we can gain explanatory power by directly considering the protein interactions themselves.

Below, I will first explain the theory and its rationale, arguing based on three postulates that it is mechanistically principled. Second, I will illustrate how I imagine the theory plays out in the context of cancer to drive its evolution, with attempts to highlight how it is matches the common trajectory of the disease and how it can accommodate diverse observations that have so far lacked explanation. Third, I will discuss the modest amount of direct empirical data that bears on the theory. Fourth, hoping to have inspired interest in testing the theory, I will offer some thoughts on how to do so.

## 2 The mechanistic basis of the permissive binding theory

### 2.1 Oncogenesis by activation

The first mechanistic postulate of the theory is that cancer is driven largely by activating functions latent in the cell.

Compared to entire organisms, cancer has an advantage in the struggle to adapt. Many of the challenges that it faces require the gain of functions – migrate, make vessels, secrete cytokines – and adaptation to ecologies – liver, bone, lymph – for which there are ready-made blueprints in its genome. Other cells in the organism, in other contexts, use these as part of their normal function. Evolution is loathe to invent from whole cloth when it can tinker around the edges (21); cancer uses the ample material of the genome, in which is contained every function performed by any cell at any time during the life of the organism.

Consider two functions already widely appreciated as key in the invasion-metastasis cascade. The first is the epithelial-mesenchymal transition (EMT). This is a programmed phenotypic shift in which epithelial cells gain the mesenchymal traits necessary for them to leave the epithelial sheet and become migratory. It comprises multiple processes, including dissolving cell-cell junctions, removing apico-basal polarity, and reorganizing the cytoskeleton. It is essential for embryogenesis, as during the concerted movement of epiblast cells into the embryonic interior during gastrulation and the long migration of the epithelial neural crest to sites around the vertebrate body. It can also be reactivated in select contexts in the adult, as in the migration of cells from the edge to the center of a healing wound. The singular term “EMT” is used across these cases despite their contextual diversity because the genetic program at their core is highly similar. This makes sense: the mechanics required are highly similar, and so evolution has not reinvented the wheel. A carcinoma cell, too, seeking to invade surrounding tissue or metastasize elsewhere, shares the initial state and required mechanics. The EMT would serve its purpose nicely, and, preformed in its genome, is low-hanging fruit. So, indeed, this same EMT is widely recognized to be at the core of carcinoma invasion and metastasis (10, 22).

A similar story is true for angiogenesis. Tumors are heavily reliant on oxygen. But carrying oxygen deep within tissue is not achievable from scratch: the only solution is to activate an existing program, latent in the genome, built for the purpose. In what has been termed the “angiogenic switch (23),” tumors do just this. Some leverage the language of their common genome to manipulate their neighbors, inducing existing vessels to expand by activating familiar angiogenic factors like VEGF (23). Others are more self-reliant, morphing themselves into vasculature with latent differentiation programs that guide them down the endothelial lineage (24). There is more than one road to Rome, but all are well-trodden in normal development.

I use these two examples because they are common and comparatively well-understood. But the principle applies generally. We do not yet know the detailed mechanisms for the many processes driving the diverse adaptations of cancer, but the intuitive stance, supported by these case studies, is that evolution is surely not re-inventing these functions when intact programs encoding them are quite literally at its fingertips.

This postulate is the least controversial, so I will not belabor it. The point for what follows is that the task facing the cancer cell is largely the activation of existing cellular pathways. The programs already exist, and cancer just needs to flip the switches.

### 2.2 Activation by binding

The second postulate is that functions latent in the cell can be activated through protein binding.

Proteins have two broad modes of function. The first I call “independent functions.” A protein acts independently when it is the direct physical executor of a change. Upstream steps involving other proteins may have been necessary to lead here, but now the protein acts under its own steam, without dependencies. These are the key effectors, typified by enzymes, like kinases that act directly to phosphorylate their targets or polymerases that act directly to synthesize nucleic acids. The second mode I call “dependent functions.” Here, a protein causes a change entirely by altering the independent activity of another protein. It may activate, repress, or qualitatively modulate this activity, changing its time, place, rate, degree, or targets. (For simplicity I will discuss individual proteins, but a rigorous application of this language refers to the minimum functional unit, of whatever size, as being independent. This accommodates the case of proteins that have no activity until they are assembled into a complex, in which all members are necessary for function: here, the complex as a whole is the unit that is independent).

An independent function can work in the absence of dependent modulation. A dependent function cannot. Its effect on the cell is expressed entirely through the independent function. Alone, it is impotent, like a transcription factor without a polymerase: utterly unable to make RNA.

The key difference for what follows is the relative difficulty of these modes. By difficulty, I mean something like the necessary level of molecular specificity. Independent functions are, in general, harder. They effect reactions that do not readily occur spontaneously. This necessitates molecular configurations that are a small fraction of the total number possible: they are “highly specific organic catalysts” (25). Such specificity entails substantial work to find this small fraction from natural selection.

Dependent functions, by contrast, can be very easy. At their easiest, they just require some amount of binding – *sticking –* to an independent protein. Sticking to an active or an allosteric site can stop a kinase from phosphorylating. Sticking near and occluding a target lysine can prevent ubiquitination and degradation. Sticking to two linker regions can bring two proteins together, making one the substrate of the other. Compared to independent functions, dependent functions have many more configurations available to them: they need not be nearly as precise.

Dependent functions are a rule, not an exception. They are everywhere, including core cellular pathways. In the cell cycle, securin acts dependently as a crucial checkpoint: it binds the active site of separase, preventing it from cleaving cohesin and driving the cell cycle until phosphorylated by Cdk1. In wnt signaling, disheveled acts dependently to activate beta-catenin, binding and sequestering the “destruction complex” that phosphorylates it and leaving it free to drive transcription. All transcription factors act dependently to increase the affinity of polymerases for their target genes. (I note here that the permissive binding theory is not limited to protein-protein interactions, but includes, for example, protein-DNA interactions, as for transcription factors).

The work of dependent functions is modest at the molecular level, but can have profound effects on the cell. They can, of course, inhibit activities: sticking to a gene’s promoter prevents its transcription. They can also activate: sticking to the active site of a kinase that marks a transcription factor for degradation *activates* that transcription factor, driving whatever cellular programs are its target. And they can invent: the binding of an activator which responds to a cue to a novel transcription factor couples transcription of its target genes and the cue, driving a new cellular response.

The point for what follows is that our intuition of what sorts of molecular interactions are required for useful cellular function often sets the bar too high. It is based on independent functions, which are the harder class, and are not representative of much work in the cell. Dependent functions mean that it is easier than we think for proteins to have useful effects. To activate, reduce, or change existing function, all that is needed is *binding*.

### 2.3 Binding by perturbation

The third postulate is that any sufficiently large perturbation to the cell can produce many new binding interactions.

We imagine protein binding as primarily “instructive.” The default state for proteins is that they do not bind to each other. They come to do so only if specifically “instructed” to by the work of natural selection.

I propose that protein binding is actually substantially (of course, not entirely) permissive. Binding is fairly common among proteins for which this has *not* been selected.

Two intuitions underlie the instructive view. The first is that it seems *unlikely* that binding would occur without having been selected for. The space of amino acid sequences is large; sampling randomly within it, approximating the absence of selection, seems very unlikely to result in sequences between which binding is favorable.

There are two errors in this intuition. The first is that proteins are not random, and especially not *with respect to each other.* They are composed substantially of domains: function-conferring subsequences widely shared across proteins. Domains drive much of protein binding (26), implying that proteins sharing domains are predisposed to share binding partners. We should expect some such binding at baseline.

The second error is more direct: even random sequences *do* bind cellular proteins. This seems surprising even to me, but I have been convinced by several recent studies showing that proteins made from random amino acid sequences can bind to cellular proteins and thereby produce essential cellular functions (27–29). The implication here is that protein binding is reasonably common at baseline, even without shared domains.

A second, stronger intuition driving the instructive view is that, because we observe fairly specific patterns of binding, the permissive view has been falsified on empirical grounds. This is incorrect because what we observe is *not* the baseline state: much binding that existed at baseline is not manifest in cells. Rather than binding being actively crafted from a default state in which it is absent, the *absence of binding is actively created from a default state in which it is common.* This is akin to synapse formation in the brain: initially abundant connections are pruned, preserving a much smaller number. This is the inverse of the standard instructive view, and is due to two mechanisms.

First, natural selection actively changes protein sequences to limit binding interactions. This could be either to remove harmful interactions per se or merely as a side effect of strengthening beneficial ones (30–32).

Second, although the “motive” for binding may exist, the “opportunity” often does not, because the proteins are never in the same place at the same time. They are expressed in different cell types, or at different times, or are localized to different places. They may inhabit the same cell, but be hogged in complexes with more abundant proteins, never free when they meet. As above, this arrangement could be due either to selection against harmful interactions per se or as a side effect of beneficial localization. Whatever the cause of their separation, offered the chance, many pairs would happily bind.

This second point predicts that forcibly colocalizing proteins should frequently produce binding not seen in normal conditions. This is just what is seen, as has been noted for years in comparing results from yeast two-hybrid screens to those from physiological pull-down or cross-linking experiments (33–35). The many “false positive” interactions found in two-hybrid experiments, while not found in normal cells, reflect something essential. Given the opportunity, proteins bind widely.

The permissive view predicts that any perturbation to the cell that changes protein abundance, *relative* abundance, localization, or affinities amounts to producing a “permissive state,” and so should produce novel binding interactions. The number of new interactions should scale with the severity of the perturbation, minor changes producing only a few, but dramatic changes producing many.

Some concrete examples may illustrate the intuition. Changes to a protein’s amino acid sequences should also tend to reverse selection’s drive to circumscribe its binding interactions, creating a permissive state. Changes to posttranslational modifications, which also perturb its physicochemical properties, should have a similar effect. Changes to transcriptional state may change protein abundance, changing binding directly by mass action; *relative* protein abundance, changing binding by competitive effects on mass action, as proteins formerly complexed with one partner become free to bind others following changes to normal stoichiometry; and localization, as proteins overflow into new cellular compartments, changing binding by bringing pairs together for the first time. Changes to protein or RNA abundance or stability, mediated by other proteins that regulate them, like ubiquitinases, RNA binding proteins, and transcription factors, should have the same effects.

I do not mean that *all* changes to these features will produce permissive states. Healthy regulation *is* change. It produces not *novel* binding interactions, but ones that have been tested and approved by evolution, either crafted for their utility or not purged because they are benign. In normal conditions, the cellular milieu is heavily controlled, constrained to this small fraction of states upon which natural selection has acted to ensure good behavior.

By contrast, perturbations to the protein repertoire that do not occur in the normal life of a healthy cell are not pre-screened by natural selection, and so are not guaranteed to be benign. The permissive baseline of protein binding here emerges, unleashing a two-hybrid experiment on a cellular scale. Interactions fall to feral baseline, and widespread binding breaks free.

I will use the term “perturbation” to refer to any change that produces a physiological state sufficiently different than those encountered in normal life that it results in a permissive state and the novel binding that results. This fits our intuitive use of the word, and emphasizes the generality that is a central feature of the theory. The possible causes of perturbations are enormous, and they are not restricted to any particular class of entity. They include internal changes, like mutations, and external changes, like chemicals, external forces, altered substrates, temperature, and much more.

### 2.4 Summary of the mechanistic basis

In brief, the permissive binding theory holds that many types of environmental and genetic perturbations produce non-physiological combinations of protein abundance and localization. It also holds that binding is – per its name – natively permissive, such that these perturbations generate a permissive state, in which emerge a large number of novel protein binding interactions. A subset of these binding interactions activate existing genetically-encoded functions, by “dependent” modes of action, that alter the activity of other, “independent” actions. A subset of these functions are those that, being useful to the cell, drive oncogenesis, and, as we will see, particularly the later stages.

## 3 The permissive binding theory at work in cancer

Having laid out the theory, I will first sketch how it might play out in the context of cancer. This is meant to be quite general, providing the spirit and shape of the theory rather than its exact details.

### 3.1 The overall trajectory of cancer evolution

Cancer initiates according to the mutation-centric framework as described in the introduction. “Driver mutations” occur, increasing proliferation and decreasing apoptosis, leading to the increased cell numbers of benign hyperplasia. These driver genes are generally pleiotropic, as evidenced by their unusually high centrality in cellular networks (36). They have roles in many cellular pathways. The driver mutations therefore have effects on the cell that extend well beyond the pro-proliferative ones for which they are selected. These include higher mutation rates, as in mutations to DNA surveillance genes like p53; and widespread transcriptional and proteomic perturbations, as in mutations to global transcription factors like N-myc, regulators of global transcription factors like CDKN2A, or central signaling hubs like Ras. As just one example, a common driver KRAS mutation (G13D) significantly changes the expression of 6,000 genes (37) and the phosphorylation state of half of all proteins (38). The side effects of driver mutations are profound: they reverberate through the cell, producing not just more growth but a broadly perturbed state.

The hyperplasia present at this stage is benign to the organism, but produces changes to tissue composition, architecture, and environment felt acutely by the hyperplastic cells. Their contact with the basement membrane is altered, changing stiffness, polarization, mechanotransductive signaling (39); their distance to nearby sources of signaling molecules is changed, giving them more or less (40); the disrupted contacts with neighbors changes those neighbors’ behaviors, making them secrete more or less or different signals (41). These environmental changes perturb the transcriptional and proteomic state of the cells.

Even at this early and benign stage, this combination of endogenous (produced by the driver mutations) and exogenous (produced by the hyperplasia) changes causes the physiology of the hyperplastic cells to be significantly perturbed. There are many changes to the numbers, locations, and states of the protein repertoire; this cellular state is now one not seen in any normal cell type. Per the permissive binding theory, this is a permissive state, allowing many novel protein binding interactions to emerge.

Per the theory, some of these new binding interactions activate latent cellular functions. These are induced “randomly,” depending only on the molecular details of the particular permissive state. There is no bias for functions useful to the cell. If no useful functions are produced, the hyperplastic process continues, bringing continual physical and environmental changes, and inducing new permissive states, new binding, and new functions.

Eventually, a function useful to the tumor emerges, providing the functions necessary for the next stage of its progression. The all-important EMT is induced, conferring the ability to invade and migrate. The relative importance of endogenous and exogenous changes in this emergence is a detail of the theory not yet clear, and will likely vary from case to case. But the exogenous ones are likely significant, as suggested by findings that changes to the stiffness of the extracellular environment can induce the EMT (42, 43) and that EMT activation varies across the spatial dimensions of the tumor, with preferential activation in cells on its edge (44).

The cancer now moves into surrounding tissue. The cells resident there are well-adapted to it and so live happily, but the invasive cells are not. For them, it is a foreign environment, and so further perturbs cellular physiology, creating permissive states. Another wave of novel binding is unleashed. New functions are generated, from which selection can pick those useful to the progression of the cancer at the present time. If the cells are in striking distance of vasculature, for example, functions that allow them to intravasate may be selected. If they are in need of new metabolic strategies, those functions will be selected instead.

Eventually, the cancer moves through circulation and enters foreign tissue. This environment, more unfamiliar than any it has encountered before, deepens physiological perturbation and creates even more permissive states. Sometimes, among the many functions unlocked are a number and kind sufficient to enable successful colonization of this new territory. A metastasis is formed.

**Figure 2:**
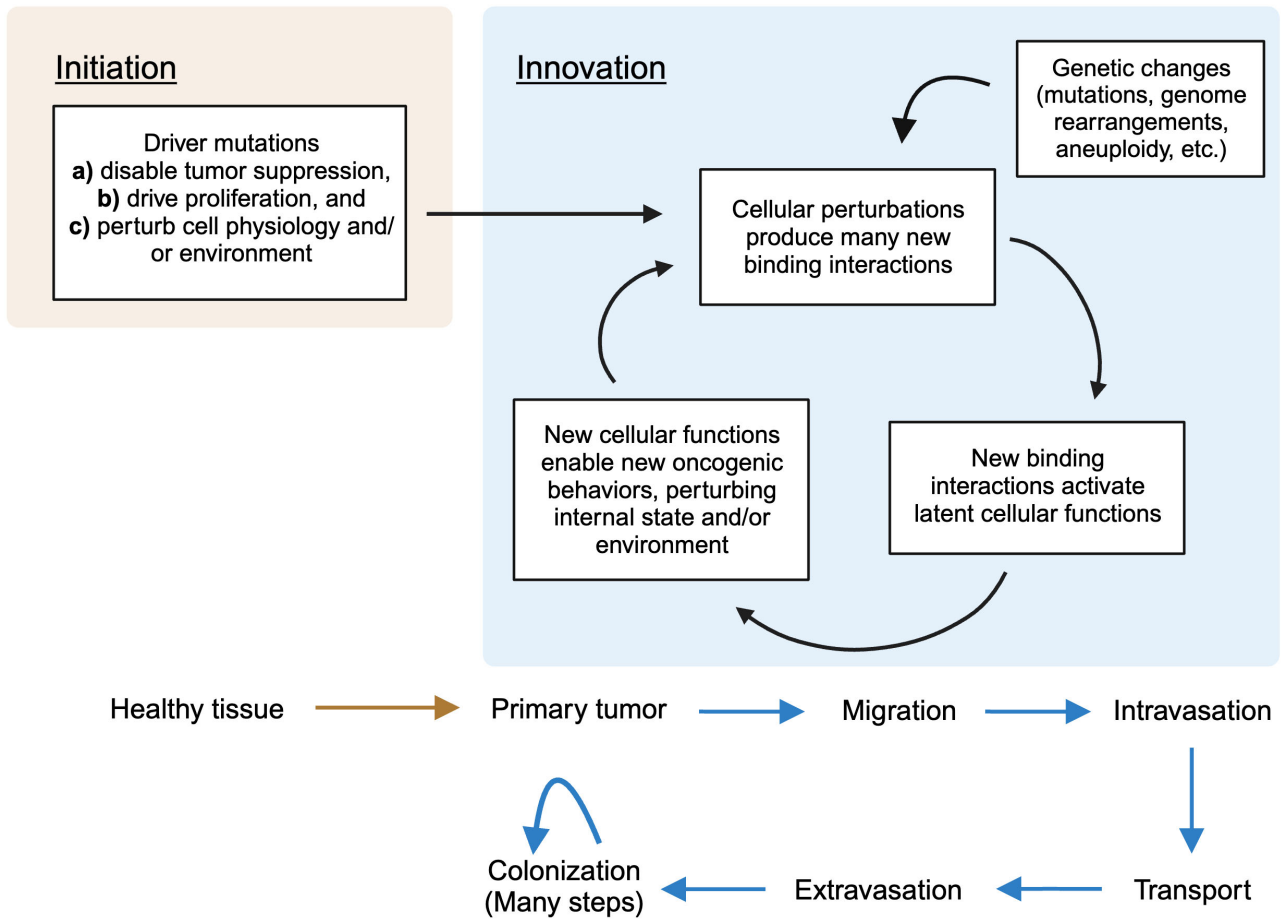
A summary of the stages of cancer evolution in terms of the present theory. In the first phase (initiation), driver mutations lead to a primary tumor by disabling tumor suppression, driving proliferation, and perturbing cellular physiology. In the second phase (innovation), an iterative process, new binding interactions are unleashed by perturbed physiology, fueling new oncogenic behaviors, which in turn cause more perturbations as, e.g. the cancer moves into a new environment. Genetic changes accumulate throughout, contributing to perturbation. (Figure created with BioRender.com).

We can briefly summarize the overall picture by delineating two phases of oncogenesis. Initiation occurs when driver mutations disable normal cellular guardrails, allowing unregulated proliferation. They also perturb the cell’s physiology internally (through disruption of pathways of which the driver genes are members) and externally (by altering the physical environment of the cells), creating the first suite of permissive states. From here, the iterative second phase, which I call innovation, begins. These permissive states create new functions; these enable access to new oncogenic phenotypes; these perturb physiology; this creates new functions – and so on. Genetic perturbations, of generally increasing severity, accumulate throughout, adding the fuel of their own resulting perturbations. A turn of this crank enables each new stage of cancer, which requires its own set of adaptations, in turn. Progression begets progression; pathology begets pathology. This summary is depicted in **Figure 2** below.

### 3.2 Other phenomenological features as explained by the theory

I will now show how the theory might be useful for explaining various other features and observations in cancer. Many other examples are possible; here, I focus on features that the standard mutation-centric picture struggles to explain or points about which readers may be wondering.

#### 3.2.1 Necessity of driver mutations

The theory holds that many different perturbations, including purely environmental ones, can create the permissive states that enable oncogenesis. Why, then do we not observe cancers triggered exclusively by environment, entirely devoid of mutations?

In normal cells, environmental insults do unleash permissive states. But the affected cells are usually then subject to physiological safeguards that, most of the time, successfully prevent excessive proliferation and cancer progression. These safeguards include processes like apoptosis and cell cycle checkpoints, and are carried out by tumor suppressor genes. Successful oncogenesis requires disabling these safeguards, which is generally possible to the extent required only through mutation of these tumor suppressor genes.

The purely environmental induction of cancer is *possible*, as has been shown in experimental conditions (45). That it does not usually occur in nature likely reflects the low likelihood of evading the tumor suppressive mechanisms that are “baked in” to normal physiology without the help of mutations.

Many diseases other than cancer cause widespread physiological perturbations, like the altered hormonal profiles and chronic inflammation of obesity and diabetes. The above argument is consistent with the observation that these diseases increase the risk of cancer (46). Lacking the key initiating mutations, these conditions alone are not sufficient to drive cancer; but, once these mutations occur, the perturbed environment that they produce increases the probability of cancer developing, and/or the speed with which it progresses.

#### 3.2.2 The role of environmental perturbation

Although environmental perturbations alone are not generally sufficient to *induce* cancer, they likely induce many of the permissive states key to its later evolution. This would explain the observed lack of characteristic metastatic mutations and the finding that they do not seem to be necessary (1, 3, 5–7). The invasion-metastasis cascade is partly or largely driven by the environmental perturbations that are rampant in these stages, rather than mutations.

The key role of environment allows the possibility that mutations within a tumor could act indirectly, by perturbing and creating a permissive state in their neighbors. The high genetic heterogeneity of many tumors (47) may reflect such a strategy.

The tumor microenvironment has increasingly been recognized as key in oncogenesis (41, 48). Some of these effects may be by way of generating permissive states in the tumor.

Why do cancers form a primary tumor before they disseminate and metastasize, rather than vice versa? Under the mutation-centric framework, this was assumed to reflect the large number of cell divisions necessary for the mutations enabling spread, an interpretation questioned by the finding that mutations do not seem to be necessary for metastasis, and that metastatic lineages can emerge quite early from primary tumors (49). The essential role of the permissive state produced by the altered physical environment in the primary tumor may be the reason.

#### 3.2.3 Stability and heritability of adaptations

If environmental perturbations cause the abilities that enable oncogenesis, can restoring a normal environment revert the cancer? This has been observed in experimental conditions (16, 17, 50).

Should this always happen? Not necessarily. A strong counterpoint is normal cellular differentiation: a heritable change in cellular state triggered by environmental cues and effected by protein interactions (often, complexes between one or more transcription factors and DNA) that is nonetheless largely irreversible.

A helpful way of conceptualizing this phenomenon is one in which the differentiated cellular state is considered an attractor in a dynamical system, stable against environmental perturbations once reached even though the inciting incident was an environmental change. An analogous model has been proposed for cancer cell states (51). If permissive states unlock new binding events that cause functions which reinforce them, the resulting positive feedback loop may form an attractor, making the cancer cell state stably heritable even when the environmental perturbation is removed. It is counter-intuitive that a non-physiological cellular state like cancer, which has presumably not been produced directly by natural selection, could be stable. But important work in dynamical systems theory has shown that stability in such systems is more common than one might think and does not necessarily require selection (52).

In the case of cancer, it may also be that the duration for which a particular adaptation is advantageous is short, such that it may not need to be as stable as is a normal cell type. For example, it may not be useful for cancers to maintain an activated EMT program after they have already migrated to a new tissue. Especially in this case, but perhaps in general, selection may be able to act to regenerate cells with useful binding interactions despite their not being stably heritable in the very long term.

#### 3.2.4 The role of transcription factors

The theory suggests a key role for transcription factors in oncogenesis. Because cancer cells likely deploy existing genetic programs (e.g. the EMT) in successful invasion and metastasis, transcription factors, as the switches that turn genetic programs on and off, are central. A new binding interaction for a transcription factor, either with a new DNA locus or with a new coactivator or corepressor, has the potential to activate a fully-formed cellular function.

There is also reason to believe that transcription factors should be uniquely vulnerable to forming new binding interactions in perturbed physiology. Many transcription factors bind cooperatively with cofactors, and many bind different targets, or have qualitatively different effects (activation versus repression) on their targets, depending on the particular cofactor to which they are bound. Small changes in the level of available binding partners can therefore have dramatic changes on target gene expression. That the relative levels (stoichiometry) of transcription factors is a major determinant of gene expression, especially in irreversible cell fate decisions, is well-known (53–56). Physiological perturbations found in cancer, with the capacity to seriously alter protein levels, might therefore be expected to dramatically change the targets of transcription factors.

The large potential of transcription factors to unlock functions useful in cancer combined with the unique vulnerability resulting from their sensitive dependence on stoichiometry suggests that they may play a special role in oncogenesis.

#### 3.2.5 Diversity of environmental insults

A dizzying variety of chemical and environmental exposures increase cancer risk. Structurally diverse chemicals; chronic inflammation; hormonal perturbation; hot liquids; infections; and more all the time (57). We now take for granted that ‘almost everything causes cancer,’ but such diversity is quite remarkable. One would like a unifying mechanism for the oncogenic activity of such varied agents. It was assumed that most carcinogens are mutagenic, but recent work has shown this not to be the case (58).

The present theory offers an answer. The only requirement to cause oncogenesis, following the acquisition of driver mutations, is any perturbation large enough to generate a permissive state. The theory is agnostic to the specifics of the causal agent. It predicts the striking generality of oncogenic activity that we observe.

#### 3.2.6 Changes in types of mutations over time

Genetic changes accumulate throughout the evolution of cancer. The new environments faced during the invasion-metastasis cascade, particularly colonization, demand a larger number, and more specific types, of adaptations than did the site of the primary tumor. The cancer must activate the specific pathways that can metabolize the new tissue’s food sources; adjust to its particular oxygen level; communicate with and coerce its new neighbors; and so on.

Increasingly, it is large, coarse, dramatic genetic changes, like aneuploidy, polyploidy, chromothrypsis, and extracellular DNA, that are useful here (14). Being of very large effect, they dramatically perturb expression levels (59, 60)to create correspondingly permissive states, creating a huge amount of new binding in one fell swoop. This ‘everything at the wall’ strategy is their best chance of hitting upon the many and specific abilities that they need.

Cells with strong tendencies to produce these dramatic genetic changes in their daughters are especially useful here. They accelerate adaptation by rapidly generating the needed variation. This may be the role of cells that are highly genetically unstable due to very abnormal karyotypes, like polyploid and polyaneuploid giant cancer cells, which have been shown to be capable of seeding metastases with much adaptive potential (12, 61).

These dramatic perturbations are selected for in virtue of the useful interactions that they produce, but also cause many useless, or even harmful, “passenger interactions.” Despite not being useful when they occur, they may become useful later, “preadapting” the cancer to new challenges. This may account for at least some of the uncanny ability of cancer cells that have adapted to one environment to adapt to another, despite the two sets of necessary adaptations not obviously having much in common.

#### 3.2.7 Tissue specificity

Because the new binding interactions that drive cancer are due to serendipitous interactions between whatever proteins were present in the perturbed cell, the theory predicts strong dependence of binding events on tissue of origin, based on its characteristic expression profile. As some perturbations are caused by mutations, some mutations should have tissue dependence, as is the case (62).

#### 3.2.8 The EMT-stemness link

It has been observed that cancer cells that have activated the EMT are also strong “cancer stem cells,” particularly capable of seeding metastases. The observation is mysterious: the EMT supports the invasion and migration process itself, but is not obviously useful for the different task of colonizing new tissue.

The present theory is consistent with this observation. It holds that any cellular state that is sufficiently dissimilar from those encountered in the normal life of a cell is permissive. The induction of the EMT does not normally occur in the cell types from which tumors have arisen, or in the environments in which they reside. Inducing an EMT program in these foreign contexts pumps its effectors into a cell teeming with proteins utterly unfamiliar to it. A large amount of novel binding is thus unleashed.

This reasoning is consistent with the additional finding that *partial* induction of the EMT, producing an intermediate state neither completely epithelial nor completely mesenchymal, causes stronger stemness than does complete induction. Extended duration of this Frankensteinian intermediate is even more dissimilar to normal physiology, and so is even more permissive.

The theory does not predict that the EMT is special in this regard. Induction of any program that is partial, in a non-physiological cell type, or in a non-physiologic environment should cause oncogenic phenotypes. Just this has been observed in a case of incomplete reprogramming (63).

The EMT is distinctive in that it is strongly selected for because of its utility for migration and invasion. Other programs will not be as often selected, and so will not have the opportunity to drive subsequent adaptation.

#### 3.2.9 New genes and cancer

If protein binding is permissive by default, and such permissive binding is prone to drive oncogenesis, then newly-born genes, for which natural selection has had limited time to prune and limit potentially harmful interactions, should be especially liable to forming such interactions during physiological perturbation, and therefore to promoting cancer.

As I noted previously in a review of so-called “*de novo*” genes, recently been born anew from noncoding DNA, this seems to be the case. Strikingly, the only known functions of human *de novo* genes are pro-oncogenic ones (64). (It was this curious observation that prompted development of the present theory).

## 4 Evidence for the theory

My discussion so far has been mostly conceptual, arguing from first principles for the mechanistic plausibility of the theory, and abductive, arguing for its ability to explain and unify much apparently disparate phenomenology in cancer. Is there direct evidence for or against the theory and its specific proposal of the centrality of novel protein interactions?

Direct tests of the theory will ideally begin with identification of binding interactions in cancer cells. This remains technically challenging at medium to large scale, and so relevant data are sparse. But there are some, which I discuss below.

It is important to note that all data discussed below are derived from cell lines, as are the vast majority of experimental data on cancer. Their applicability to the primary aspiration of the theory – the invasion-metastasis cascade – is therefore limited. Although developed primarily to answer the puzzle of invasion and metastasis, there is nothing preventing the theory from operating in earlier cancer, as described above; so I take these results to be promising overall.

### 4.1 Many novel protein interactions in cancer cell lines

A basic prediction of the theory is that there should be many new binding interactions in cancers. Are there data to this effect?

An important kind of binding interaction is between proteins (as opposed to between, for example, proteins and DNA, as for transcription factors). There is much work aiming to indirectly infer protein interaction networks in cancer lines (from, for example, combinations of transcriptional and genomic data), which has generated results suggestive of many new interactions (65–67). While these results are interesting support for the theory, I will focus here on direct experimental tests, which seem to me more decisive.

One recent pair of studies in a) head and neck (68) and b) breast cancer (69)cell lines systematically identified all binding partners for 30-40 proteins known to drive their respective condition (but not all mutated in the cell lines used). For each cancer type, these protein interactions were identified in two independent cancer lines and, as comparison, in one non-cancerous line from a matched normal tissue.

**Figure 3:**
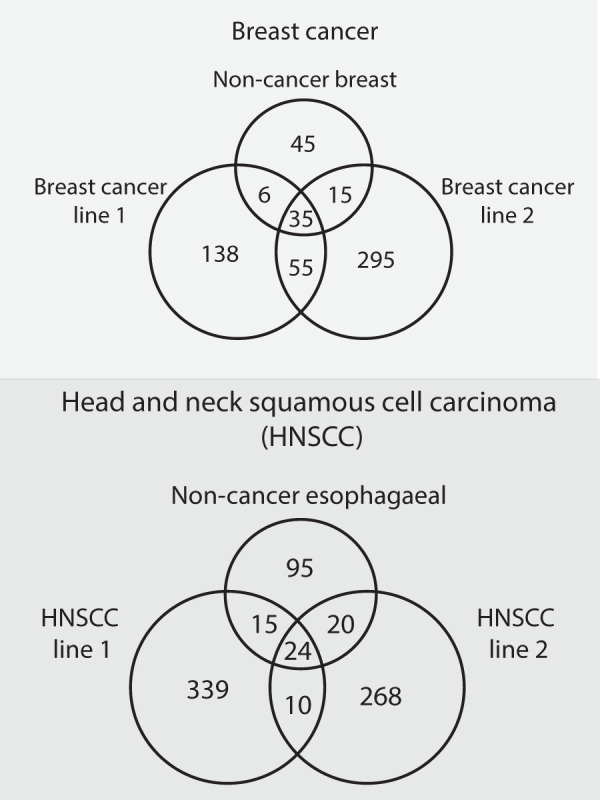
The results of two studies (68, 69), that identified protein-protein interactions in cancerous and non-cancerous cell lines. For each of two types of cancer (breast cancer and head and neck squamous cell carcinoma), two cancerous cell lines and one non-cancerous cell line of matched tissue type were profiled. The interactions found in the three lines of each cancer type were then compared to determine how many were shared. The number of unique and shared interactions in each line are depicted in the Venn diagrams.

The number of protein-protein interactions identified in each cell line, and the number found to be shared between them, is shown as **Figure 3** below. Two features are striking.

First, all four cancer lines have many more protein-protein interactions, by 2-4 fold, than their non-cancerous counterparts.

Orthogonal support for this conclusion comes from a different study (which, because it took a somewhat narrower and less physiologically relevant approach, I give less primacy). 32 driver mutations were engineered into non-cancerous (HEK293T) cell lines, and interactions between each wild-type and mutated protein and a defined set of ~550 other proteins were identified. The mutated proteins had many more interaction partners (340) than the wild-type.

It is not surprising that cancer cells, being different than normal cells, should have some different interactions. It is less obvious that there should be quite *so many* differences. The addition of new interactions numbering *twice as many as the original total* is profound.

It is also surprising that, in addition to there being many *new interactions*, the *total number* of interactions is so much higher in cancer cells. It might have been that cancers gain and lose a similar number, roughly maintaining the total. This is not what we observe. The large increase in the total number of interactions is specifically consistent with the hypothesis of permissive binding. Interactions are abundant at baseline, pruned in normal conditions, and returned to baseline by perturbation.

Second, within a cancer type, relatively few of the new interactions in each cancer line are shared with the other.

This too is consistent with the hypothesis of permissive binding. Perturbations cause permissive states, but different ones: they allow interactions between whatever proteins are present in the cells, determined by their history, their environment, and the precise nature of the perturbation. Different cancers, differing in all of these features, should have different sets of interactions.

The intersection of these interactions may be the key “drivers” of oncogenic function in these cancers, much as we infer that genes recurrently mutated in many independent tumors are the genetic drivers. The others may, similarly, be “passenger interactions”.

### 4.2 New binding interactions may be the root of differential dependencies

A second kind of evidence comes from experiments identifying genes upon which cancer lines have become dependent during their evolution. This is done by systematically knocking genes out or down, one at a time, and determining which are essential for the survival of the cancer line, but not for normal cells (70).

This work has made two points clear. The first is that there is substantial variation between cancer lines in which genes have become essential, a phenomenon termed “differential dependence.” The second is that, while some of the differential dependencies in a given cancer can be explained by the mutations that it bears (for example, gain-of-function mutations to canonical driver genes often result in their becoming essential in “oncogene addiction”), many cannot. One recent analysis of 769 cancer cell lines found 550 genes with differential dependence, of which only 127 – less than a quarter – could be explained by mutations. Adding the cancer type in question to the analysis increased power, allowing 227 of the 550 dependencies to be accounted for (71)– still less than half.

Differentially dependent genes are promising as a clue to the basis of the new abilities gained by cancer cells. Because their role differs dramatically between cancers and their ancestor, they may be the key substrates of change.

In the structure of the permissive binding theory, differentially dependent genes are well-suited to be the proteins directly participating in the novel binding interactions. They are essential specifically in cancer and not in normal cells, due to the functions that they newly drive in cancer. They differ between cancer lines, as new interactions should, having been generated by idiosyncratic endogenous and exogenous perturbations. They are not robustly predictable from mutations, as they result from a wide variety of complex genetic and environmental perturbations. They are much *more* predictable from mutations with the additional context of cell type, which dictates what proteins were present and thus available for binding when the perturbation occurred.

Are differentially dependent genes those involved in the new binding interactions found in cancers? Sadly, there are no experimental data to this effect. But we can ask whether there are known features of these genes suggesting their tendency to be involved in binding interactions in general. A gene ontology analysis of the differentially dependent genes identified in the above analysis reveals that they are remarkably enriched for binding ability. The 5 most statistically significant category enrichments (having removed “molecular function,” which, since it is the meta-category selected for use in the analysis, strikes me as trivial) are reproduced (71) as **Table 1** below.

**Table 1:** Results from (71), which first identified “differentially dependent” genes, defined as genes whose essentiality (as determined by causing reduced proliferation when knocked out or down) varies across many cancer cell lines, and then performed a gene ontology (GO) enrichment analysis to determine whether particular functions were enriched among these genes. Shown are the top five most statistically significant GO categories resulting from the analysis.

GO molecular function	Number	Expected number	P-value
Protein binding (GO:000515)	468	361.15	7.3x10^-29^
Binding (GO:0005488)	491	415.98	7.2x10^-21^
Enzyme binding (GO:0019899)	135	57.57	1.4x10^-20^
Protein domain specific binding (GO:0019904)	65	17.83	3.3x10^-18^
Kinase binding (GO:0019900)	67	19.27	8.8x10^-18^

The three categories with highest fold enrichment (rather than the lowest p-value; all still statistically significant) are “C-X3-C chemokine binding,” “BH3 domain binding,” and “alpha-catenin binding.” And, of all 109 GO categories found to be significantly enriched, 73 (67%) include the term “binding,” compared to only 1878/11236 (17%) of all categories.

### 4.3 A putative example of the theory in action

There have to my knowledge been no systematic searches for new protein binding interactions that drive the evolved functions essential for cancer, but a beautiful example has been discovered incidentally (72), which I describe below.

Beta-catenin is a transcriptional coactivator that commonly contributes to early oncogenesis. In its canonical role, it is activated in response to wnt signaling, where it acts dependently to displace repressors from transcription factor TCF4, allowing transcription of its target genes. Some colon cancers become dependent on beta-catenin activity following its activation early in their evolution. For example, as shown in **Figure 1**, mutations reducing the activity of APC, which sequesters beta-catenin, are frequent.

What is the molecular mechanism behind the essential beta-catenin activity in these cancers? One might expect that activation of beta-catenin increases its activity within its normal TCF4 pathway. Surprisingly, a systematic screen for essential genes in these lines failed to find TCF4 pathway genes. Instead, they found an unexpected dependence on transcriptional coactivator YAP1, canonically involved in Hippo signaling, and transcription factor TBX5. These proteins were shown to assemble into a complex that transcribes antiapoptotic genes including BCL2L1, promoting survival. All three were shown to be necessary for this anti-apoptotic activity and for tumor growth *in vivo*.

Three points are important for our discussion.

First, this is an exquisite case study demonstrating the centrality of a new binding interaction in the survival of a cancer: rather than merely causing more activity in its standard TCF4-related role, beta-catenin forms a new complex containing TBX5 and YAP1. It is relevant to the above data to note that this new binding interaction is the molecular basis of a differential dependence: other colon cancer lines were not dependent on this new complex.

Second, this binding interaction produces function precisely in the manner predicted by the theory. It turns on dependent functions: beta-catenin acts dependently, binding to a coactivator and a transcription factor to bring the transcription factor to DNA, where it activates the existing cellular program of BCL2L1-mediated suppression of apoptosis.

Moreover, the formation of the novel complex was likely dependent on perturbations induced by the driver mutation or subsequent environmental perturbations. Here is a plausible scenario. Following activating mutations, for example to the APC, beta-catenin is more abundant in the cytoplasm. It has a latent propensity to bind YAP1: it does so in the very different conditions of heart development (73), but, even though YAP1 is expressed in most epithelia (72), is normally prevented from doing so in colon cells. The increase in beta-catenin creates the permissive conditions that permit the interaction in this foreign context. Either because this association changes its localization or physicochemical properties, or because of other perturbations to the cell, YAP1 then permissively binds TBX5. Again, it does not normally do so; but, again, it has a latent ability, as shown by experiments in which their binding results from their overexpression (74). The YAP1-TBX5-beta catenin complex is now formed, and binds to the promoters of anti-apoptosis genes. Here again this is not the normal function of the transcription factor TBX5, but again it has latent ability to do so, having been shown to bind these genes when overexpressed (75), and it is likely aided by new binding partner YAP1, which is a coactivator for these targets in other conditions (76). Later in the evolution of such cancers, loss of beta-catenin’s normal binding partner TCF4 can further increase the amount available to join this complex, explaining why TCF4 loss promotes tumor progression (77), an observation that, when TCF4 itself was thought to be co-complexed with beta-catenin as part of the key oncogenic complex, was difficult to explain.

**Figure 4:**
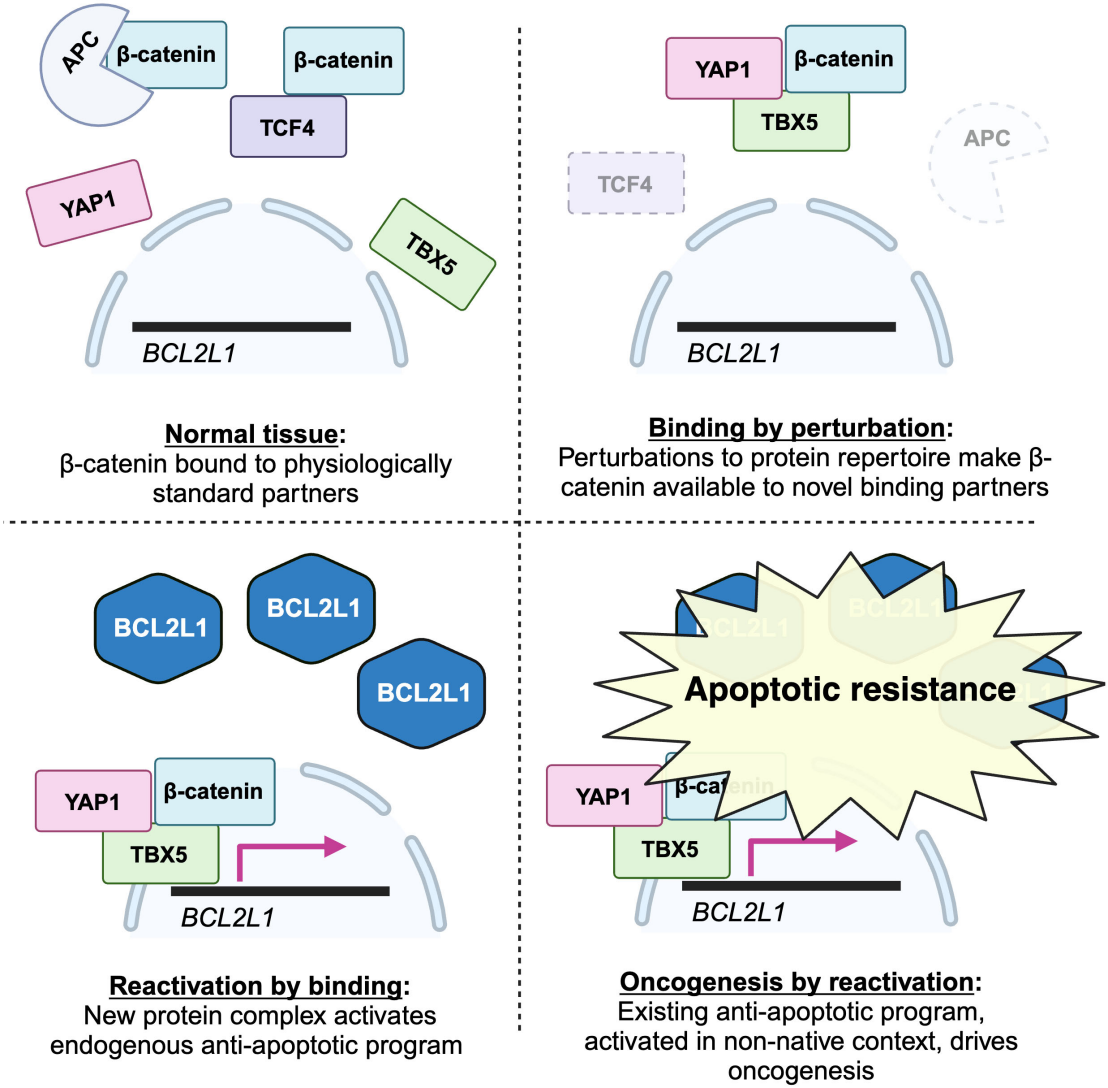
A graphical depiction of my interpretation of the results in (72) within the framework of the permissive binding theory. The first event in colorectal cancer progression occurs when deletion or disruption of the APC, a canonical driver mutation, perturbs the cell by decreasing the levels of normal interaction partners of beta-catenin. This leaves beta-catenin free to form new binding interactions with non-standard partners TBX5 and YAP1. Once this novel complex is formed, it can bind to promoters of anti-apoptotic genes like BCL21L, which beta-catenin does not regulate in normal physiological contexts, and drive their expression. This switches on the anti-apoptotic program, which, repressed in normal physiological conditions, confers the new ability of apoptotic resistance on the developing cancer. (Figure created with BioRender.com).

Though not discovered with the theory in mind, this looks enormously like a demonstration of it. Here is a case in which new binding interactions, enabled by perturbations to the cell, activate an existing cellular program, driving the differential dependency that reflects this cancer-specific adaptation. A graphical summary showing a simplified version of these findings, and their casting in terms of the mechanistic steps postulated by the theory, is shown below in **Figure 4**.

Stumbling across a phenomenon at first thought to be unusual has often been the tip of the iceberg, preceding realization that it is actually pervasive. Examples like this may be the rule rather than the exception.

### 4.4 Summary of the available data

The existing data indicate three things. First, that a new binding interaction underlies at least one ability – the repression of apoptosis – crucial for the evolution of one cancer, through molecular mechanisms consistent with those predicted by the theory. Second, that there are many new binding interactions present in cancers. Third, that genes responsible for a large number of essential cancer-specific abilities are enriched for known functions related to binding. These, to my mind, form a promising initial basis for belief in the utility of the theory, warranting further testing.

## 5 Testing the theory

An essential feature of the permissive binding theory is that it makes concrete and testable predictions. These are:

1. Cancer cells have more protein interactions than non-cancerous cells.
2. A subset (of unknown size) of these cancer-specific protein interactions cause cancer-specific phenotypes, such as invasion, metastasis, colonization, etc.

Prediction 2 is in many contexts the most important consequence of the theory, but could be true for reasons other than the specific mechanism of permissivity proposed here, which is tested by prediction 1.

Small-scale tests of prediction 1, including the studies discussed above, have already been performed, but larger-scale validation should follow. This requires systematically identifying the binding interactions in many cancers (and non-cancer controls). In an ideal world, this would be done for a variety of cancer types; for primary tumors and especially metastases, rather than cell lines (this is especially important given the importance that the theory places on environment, which will not likely be exactly recapitulated in cell culture); and at proteome-wide scale. Realistically, the limitations of existing technology make many of these desiderata difficult; studies in cell lines focusing on a subset of proteins may be more realistic. To be maximally informative within these constraints, the number of cell lines should be sizable (more on this below), with multiple lines per cancer type and multiple cancer types represented, and the selected ‘bait’ proteins should be diverse along the axes of function (including receptors/signal transduction machinery, intracellular signaling hubs, transcription factors, etc.) and known oncogenicity (canonical driver proteins and those not as clearly implicated in cancer). Techniques like those used in the analysis of breast cancer and HNSCC lines discussed above (68, 69), as well as many other types of approaches for which there are proof-of-principle demonstrations (66), may be useful here.

Prediction 2 can be tested directly or indirectly. Direct tests can be achieved in two general ways. The first is to *disrupt* a subset of the interactions identified in prediction 1 and assessing whether, and which, oncogenic properties of the cells are affected. Disrupting protein interactions is known to be difficult, and the best strategy will likely depend on the particular interaction in question. For example, there is a growing repertoire of existing small molecule interaction inhibitors, especially for proteins already of therapeutic interest (e.g. Kras (78)); although made to target specific protein pairs, they could also affect interactions between those proteins and other, new partners. New inhibitors could also be sought via standard screening approaches. Genetic approaches, if a particular binding site is known or likely given existing knowledge, may also be possible. The second direct test is the complementary approach: *inducing* a particular protein interaction in a cell line lacking the oncogenic phenotype of interest and determining whether the interaction confers that phenotype. This strikes me as the more powerful approach, as it minimizes the probability of misleading side effects from the perturbations suggested above. The task of inducing protein interactions is also challenging, but again, the field is progressing fairly rapidly on this front (79, 80). As a true gold standard for causality, as is the case in molecular genetics, one might hope to employ both the perturbation and induction methods in combination: perturbation followed by induction in the same cell line would function analogously to a “knockout” and a “rescue” experiment, which would be strongly compelling. Once an interaction is disrupted or induced, standard *in vitro* and *in vivo* assays for particular oncogenic phenotypes (like wound closure assays for migration, transwell assays for invasiveness, and transplant assays for metastasis (81)) can be used to assess the effect on phenotype.

An indirect test of prediction 2 may be simpler, as can be seen by analogy to the history of the mutation-centric picture. The hypothesis of the causality of mutations in early cancer turned out not only to be correct, but to be realized in a simple form. Rather than each case of cancer evolving by way of its own unique, *idiosyncratic* set of mutations, there is widespread convergence across cancers, both within and between types, resulting in *shared* mutations. When sequencing revealed these shared mutations in cancer after cancer, much more often than could be explained by chance, they were immediately implicated as causal, even in lieu of direct experimental data. Analogously, if the causal interactions posited by prediction 2 include some that are recurrently converged upon by many different cancers, these shared interactions should be found in cancers more often than is expected by chance, and so should be identifiable with statistical testing of datasets generated from prediction 1.

Here is one possibility for such a test. We consider a null model in which the perturbations that characterize cancer probabilistically cause some set of **I** possible new protein interactions, but that none of these is beneficial in cancer progression, such that each cancer acquires some number from this set at random. In this model, the only reason that an interaction would be found in multiple cancer lines is by chance; if we find interactions that occur more frequently than this chance level, we may suspect that they are causal. Suppose that we identify interactions in N total cancers. Under this model, the probability of finding any particular interaction in one of the cancers **C**, which has acquired **C_m_** interactions from the possible set of **I** interactions, is P(C) = **C_m_/I**. The number of total cancers, **T**, in which a particular interaction appears is distributed as the sum of N independent Bernoulli distributions, one for each of the N cancers, with cancer-specific parameter **p_m_** = **C_m_/I**. This distribution is also known as the Poisson binomial distribution, and although its analytical form is complex, per the Lyapunov Central Limit Theorem, it can be well-approximated by a normal distribution at even modest values of N, with the same mean and variance as given by the standard sum of its component distributions (82). We can therefore estimate the total number of cancers T in which we expect to find a particular interaction under this null model by a normal distribution with mean $\sum_{m=1}^{N}p_{\text{m}}$ = $\sum_{m=1}^{N}C_{\text{m}}$ /I and variance $\sum_{m=1}^{N}p_{\text{m}}$ (1-p_m_) = $\sum_{m=1}^{N}C_{\text{m}}$ /I(1-C_m_/I).

What should we use for the number I, the total number of possible interactions from which cancers sample independently? A strict upper bound is the total number of interactions in the human proteome, the square of the number of human proteins, ~4x10^8^. Realistically, this is vastly too high: many interactions will likely not be detectable even if present due to low abundance or other technical limitations, and many are likely simply not possible to generate even in a perturbed cell. Suppose that in our **N** cancers, we find a total of **U** unique protein interactions. We might use **U** as the value of **I**. This is obviously an underestimate, but an underestimate has the nice property of making for a conservative statistical test, increasing the probability of convergence under the null model.

We can use this model to perform a rough power calculation: how many lines would we need to profile to see significance? This depends on the details of the above parameters, but using numbers on the order of those from **Figure 2**: if we have 10 cancers, with 200 interactions each, and only mild overlap between them, such that U is 1800 total interactions, we would be able to detect statistical significance at p=0.05, including multiple test correction, for interactions found in 6 or more cancers. If we increase the number of cancers, but hold constant each cancer’s number of interactions and the overlap between them, with 20 cancers, we can detect significance at 0.05 for interactions found in 7 or more. We will likely want to include more cancers than this merely for the sake of including a diversity of types; this is merely meant to demonstrate that the prospect of gaining evidence for causality is feasible from the standpoint of statistics.

As realists, we must admit that there is likely to be some dependence on cancer type, as we see for mutations, and on metastatic site, as colonization of different tissues likely requires different abilities. But there may be some complexes that transcend these features, corresponding to functions needed in most or all metastatic sites (e.g. for immune escape). Convergence within these categories seems likelier, but will increase the risk, for which there is not as clear a counterpart in the mutation-centric picture, that shared environmental features rather than causality produce the shared interactions. The ideal scenario is that most interactions are not shared between cancers, suggesting that the above null model is generally a reasonable one, and that a few standouts are widespread. This is more or less what is seen in **Figure 2**: most interactions are not shared between the cancer lines, suggesting that there is not widespread convergence due to environment alone.

A negative result would not disprove the theory; it could merely be that interactions are idiosyncratic rather than shared. In this case, experimental perturbations, as described above, will be necessary.

## 6 Conceptual and practical features of the theory

I will now mention two features of the theory that are not related to its probability of being correct, but that are of conceptual and practical interest respectively.

### 6.1 Compatibility with other theories

An important difference between the present theory and many alternatives is that it includes a causal picture that identifies not only the *ultimate* causes of oncogenesis (here, initiating mutations or environmental perturbations), but its *proximal* causes: novel binding interactions. Beyond merely including both, the theory is primarily concerned with, and most specific about, this proximal cause. It is essentially agnostic on, and can accommodate wide variation in, the identity and relative importance of ultimate causes.

By contrast, most alternatives are hypotheses about ultimate causes. For example, epigenetic theories may invoke changes in DNA methylation or histone modifications. These leave unspecified the details of *exactly how* these changes produce oncogenic functions.

This difference in level of specification means that many alternative theories can be subsumed without modification under the permissive binding theory. For example, changes to DNA methylation may perturb transcription, leading to imbalances in the levels of proteins present in the cell. In the language of the present theory, DNA methylation may perturb the cell to create a permissive state. The same subsumption can be performed for less mainstream theories, like the genome architecture theory (83), which proposes that oncogenesis is due to dramatic and rapid genome rearrangements, or the tissue organization field theory (84), which proposes that oncogenesis is due to microenvironmental perturbations in the surrounding tissue.

All of these theories may be true to some degree. The permissive binding theory offers a unifying substrate onto which these diverse causes converge in producing their effects. This substrate – protein binding – is well-positioned to both explain and predict the observed effects, being the direct effector of cellular functions.

### 6.2 Implications for cancer therapy

The theory clearly suggests that targeting the novel protein interactions that cause key oncogenic phenotypes may be of therapeutic benefit. Binding interactions are notoriously difficult to target with small molecules (85), to the extent that they have been referred to as “undruggable” (86), but recent work on the problem, has shown promise (87).

That targeting protein-protein interactions could be a fruitful therapeutic avenue in cancer is not a new idea (88). The permissive binding theory merely emphasizes their importance, arguing that, because they are the most directly causal alteration in cancer cells, they are the single best point of attack.

Importantly, the permissive binding theory also suggests that the causal interactions in cancer are *novel* ones, not present in non-cancerous cells of the same tissue. If true, it is possible that side effects to therapeutics targeting these interactions will be less profound than those resulting from other therapies, which target pathways and mechanisms also found in normal cells.

## 7 Discussion: hope for simplicity

The permissive binding theory is similar in shape to the mutation-centric picture. Both posit discrete entities (mutations, binding interactions) that change during the evolution of a cancer to directly produce oncogenic functions. But, as we have seen, the consequences of taking protein interactions as the central object in a framework of cancer are quite different than those implied by mutations alone. I think it is better-suited than the mutation picture to explain the invasion-metastasis cascade on conceptual grounds, and am encouraged by initial empirical support. The theory’s biggest weakness is that this support is limited to the very small amount of available data that are suited to test it. I hope that this analysis inspires further tests, including but not limited to those suggested above.

In some ways, the permissive binding theory differs from the mutation-centric picture primarily in its esteem for the complexity of biology. It does not limit itself to a picture in which the cell operates akin to clean cartoons in textbooks, in which proteins interact only with their designated playmates, proceeding directly to them and carrying out their jobs in deterministic sequences. It opens itself to one that is more realistic to the physical and evolutionary mechanisms that undergird living systems: movements and interactions of proteins are stochastic, and the cell is only exact as it needs to be, dictated by the normal physiological contexts upon which natural selection primarily acts.

It might also be thought of as differing in accepting the limitations of our knowledge. Proteins are in most cases the final output layer of the cell: they are what makes biology go. The causes of phenotypes must, in the end, be descriptions of *what proteins are doing.* In an ideal world, we would be able to input state variables of any cell – its mutational repertoire, its physical environment, its transcriptional state, and so on – and output what its proteins are doing. But we live far from this ideal world. The permissive binding theory accepts this reality, relinquishing hope of being able to squint through the narrow lens of mutations and accepting the need, more technically difficult though it may be, to look to the proteins themselves to tell us what they are doing.

The permissive binding theory is here discussed in the case of cancer, but has applications beyond it. It may underlie pathological mechanisms in chronic diseases in which the physiological environment is altered as either cause or consequence, like inflammation, diabetes, and cardiovascular disease. Moreover, it is primarily an evolutionary theory, developed to address the evolutionary puzzle of invasion and metastasis, which it does by positing generic perturbations as a source of “unlocking” new functions. This offers a general mechanism that may be at play in other evolutionary contexts.

The permissive binding theory proposes that the waters of our attempts to understand cancer evolution have appeared so muddy because we have focused on the wrong entities. It offers a different level of analysis, at which formerly disjointed observations and mechanisms may collapse into a coherent whole. It is well-positioned to do this, being a claim about the interactions between proteins, which are the final effector of cellular behavior, and so are a natural point of convergence for observations regarding varied underlying mechanisms. Heterogeneous molecular changes, varied in kind and inconsistent in occurrence, that characterize cancers – point mutations, epigenetic mutations, aneuploidies, polyploidies, extrachromosomal DNA, transcriptional changes, translational changes, posttranslational modification changes, metabolite changes, morphological changes – may converge in their effects on the proteins: by different mechanisms, they may produce the same few key complexes.

I think the theory’s biggest virtue is that, if it is true, things could turn out to be simpler than they now appear (89).

## Data availability statement

Publicly available datasets were analyzed in this study. This data can be found here: https://www.ncbi.nlm.nih.gov/pmc/articles/PMC8979493/bin/mmc4.xlsx.

## Author contributions

CW: Conceptualization, Writing – original draft, Writing – review & editing.
